# *SIRT1* rs2394445 associates with serum triacylglycerol lipidomic profile in metabolic dysfunction-associated steatotic liver disease

**DOI:** 10.3389/fnut.2026.1835298

**Published:** 2026-07-22

**Authors:** Qin Pan, Can-Jie Guo, Hai-Xia Cao, Rui-Dan Zheng, Yu-Qiang Mi, Rong Zhang, Feng Shen, Qing-Yang Xu

**Affiliations:** 1 Shanghai Key Laboratory of Pediatric Gastroenterology and Nutrition, Xin Hua Hospital, Shanghai Jiao Tong University, School of Medicine, Shanghai Institute for Pediatric Research, Shanghai, China; 2 Department of Gastroenterology & Endoscopy, Xin Hua Hospital, Shanghai Jiao Tong University, School of Medicine, Shanghai, China; 3 School of Health Science and Engineering, University of Shanghai for Science and Technology, Shanghai, China; 4 Department of Gastroenterology and Hepatology, Ren Ji Hospital, School of Medicine, Shanghai Jiao Tong University, Shanghai Institute of Digestive Disease, Shanghai, China; 5 Diagnosis and Treatment Center for Liver Diseases, Zheng Xing Hospital, Zhangzhou, Fujian, China; 6 Department of Infective Diseases, Tian Jin Infectious Disease Hospital, Tianjin, China; 7 Graduate School, Henan Medical University, Xinxiang, Henan, China; 8 Department of Gastroenterology, Shigatse People’s Hospital, Shigatse, Tibet, China; 9 Department of Gastroenterology, Tai Zhou Hospital of Zhejiang Province Affiliated to Wenzhou Medical University, Linhai, China

**Keywords:** lipidomics, metabolic dysfunction-associated steatotic liver disease, silent information regulator 1, steatosis, triacylglycerol

## Abstract

**Aims:**

This exploratory study aimed to identify the effects of *SIRT1* polymorphisms on the serum lipidomic profile and pathological characteristics in metabolic dysfunction-associated steatotic liver disease (MASLD).

**Materials:**

Chinese Han patients with biopsy-proven MASLD were genotyped for the single nucleotide polymorphisms (SNPs) in *SIRT1*. Lipidomics based on untargeted ultra-performance liquid chromatography–tandem mass spectrometry (UHPLC-MS) was applied to map the *SIRT1*-driven serum lipid signature, followed by correlation analyses linking these lipid traits to hepatic steatosis, ballooning, lobular inflammation, and fibrosis. Integrative pathological scoring finally explored how *SIRT1* variants that remodel circulating lipids may be associated with MASLD-specific histological change.

**Results:**

*SIRT1* SNPs (rs3740053, rs2394443, rs2236318, rs7896005, rs2273773, rs2394445, and rs2394446) demonstrated extensive association with serum lipidomics of MASLD patients. *SIRT1* rs2236318, rs7896005, and rs2394445, affecting 2, 7, and 7 serum triacylglycerols (TAGs) respectively, dominated the *SIRT1* SNP-lipidomics association. Quantitative analysis revealed significantly elevated TAGs levels (48:0, 50:0, 50:1, 52:0, 52:1, 52:2, and 54:1), the most abundant species, in MASLD patients with rs2394445 A/A genotype compared with A/T+T/T. These elevated rs2394445-dependent TAG levels were associated with high-grade hepatic steatosis (rho: 0.367 to 0.540, *p* < 0.033–0.001) rather than ballooning, lobular inflammation, or fibrosis. The significant correlation of rs2394445 A/A genotype with severe steatosis suggested a potential pathological link through TAG profile modulation. In contrast, rs2394445 showed a limited effect on total circulating lipids and obesity indices (BMI, WHtR).

**Conclusion:**

The A/A genotype at *SIRT1* rs2394445 is associated with elevated serum TAGs containing saturated or monounsaturated fatty acids, which are linked to high-grade hepatic steatosis in MASLD independently of obesity.

## 1 Introduction

Metabolic dysfunction-associated steatotic liver disease (MASLD) represents a recently redefined nosological framework, strategically repositioning metabolic dysfunction as the central pathophysiological driver (1, 2). This umbrella terminology encompasses the entire histopathological continuum, ranging from metabolic dysfunction-associated steatotic liver (isolated hepatic steatosis) through metabolic dysfunction-associated steatohepatitis (MASH), which may subsequently advance to liver fibrosis, cirrhosis, and ultimately hepatocellular carcinoma (1, 2). From an epidemiological perspective, MASLD constitutes the most prevalent chronic liver disease globally. Pooled prevalence estimates indicate that 32.4% of the worldwide population is affected. Notably, the global prevalence of MASLD has increased markedly from 25.5% before 2005 to 37.8% after 2016, with an overall incidence of 46.9 cases per 1,000 person-years and marked male predominance (70.8 in men versus 29.6 in women) (3).

Accumulating evidence has indicated that dyslipidemia is not only a prerequisite for the diagnosis of MASLD (4), but also forms a bidirectional, causal loop with the individual components of metabolic syndrome, thereby indirectly fueling its development (4, 5). Beyond the well-recognized effects of Western dietary patterns and sedentary lifestyle, genome-wide and candidate-gene studies have identified single-nucleotide polymorphisms (SNPs), such as *PNPLA3*, *APOE*, and *APOC3*, *etc*., as key genetic determinants that modulate the susceptibility to dyslipidemia-based MASLD (6–8). The *PNPLA3* rs738409 G allele confers a higher risk of hypertriglyceridaemia, especially in Asian populations, while promoting intra-hepatic lipid accumulation and progression from simple steatosis to nonalcoholic steatohepatitis (6). Likewise, *APOC3* variants rs651821 and rs2854116 attenuate lipoprotein lipase activity, leading to elevated triglyceride concentrations and an increased risk of MASLD that exhibit ethnic-specific distributions (7) The *APOE* ε3/ε3 genotype enhances susceptibility to MASLD, but the ε3/ε4 haplotype is protective. Intriguingly, ε4 elevates systemic dyslipidaemia risk, yet curtails hepatic steatosis *via* divergent effects on VLDL and HDL metabolism (8). Moreover, abnormal lipidomic characteristics may underlie the effect of dyslipidemia on MASLD (9, 10).

Recently, genetic variants of silent information regulator 1 (*SIRT1*) exhibit intimate associations with the predisposition to metabolic diseases (11–17). A case–control study in Bangladeshi subjects has revealed that the rs3758391 polymorphism significantly elevates the risk of type 2 diabetes mellitus (T2DM) under a codominant model (TT versus CC) (11). In Pima Indians, two tag SNPs (rs10509291, rs7896005) demonstrate associations with T2DM alongside diminished acute insulin response to intravenous glucose challenge (12). In addition, T2DM patients carrying the CC genotype at rs7069102 display increased susceptibility to diabetic nephropathy across both codominant and recessive inheritance patterns (13). While rs33957861 and rs11599176 correlate with adult obesity, carriage of the rs7895833 G allele confers heightened risk for childhood obesity (14, 15). Furthermore, the rs1467568 G allele associates with a reduction in hyperbetalipoproteinemia prevalence, though rs7895833 conversely links to dyslipidemia (16, 17). However, the SNP-specific effects of *SIRT1* on serum lipidomic profiles and their interaction with MASLD pathophysiology remain poorly elucidated.

We, therefore, performed an exploratory study using *SIRT1* genotyping to categorize Chinese patients with biopsy-confirmed MASLD, applying ultrahigh-performance liquid chromatography–tandem mass spectrometry (UPLC-MS/MS) to profile serum lipidomic alterations associated with *SIRT1* polymorphisms. Correlation analysis subsequently explored connections between *SIRT1*-related lipid metabolites and liver histopathological features. Ultimately, variant-specific scoring of steatosis, ballooning, inflammation, and fibrosis was conducted to illuminate the potential influence of lipidomics-affecting *SIRT1* variants on disease-specific pathological characteristics.

## 2 Materials and methods

### 2.1 Study population

MASLD patients were consecutively enrolled from three tertiary centers (Xin Hua Hospital, Shanghai [*n* = 17, 50%]; Tian Jin Infectious Disease Hospital, Tianjin [*n* = 9, 26.5%]; Zheng Xing Hospital, Zhangzhou, Fujian [*n* = 8, 23.5%]) between January 2012 and June 2013 using identical inclusion/exclusion criteria and standardized biopsy protocols across all sites. No center-specific selection biases were present. Serum specimens for lipidomic profiling were obtained following a 12-h overnight fast on the same day as the liver biopsy procedure, immediately aliquoted, and stored at −80 °C until UPLC-MS/MS analysis.

The contemporary diagnostic criteria for MASLD stipulate the coexistence of hepatic steatosis and at least one cardiometabolic risk factor. The presence of overweight or obesity constituts a major qualifying criterion, defined as body mass index (BMI) ≥ 25 kg/m^2^ (≥23 kg/m^2^ in Asian populations) or increased waist circumference (>94 cm in males, >80 cm in females). Fasting plasma glucose ≥5.6 mmol/L (≥100 mg/dL), 2 h-OGTT ≥7.8 mmol/L (≥140 mg/dL), HbA1c ≥ 5.7%, established T2DM, or ongoing glucose-lowering therapy satisfies the diagnostic threshold. Other criteria include established by systemic blood pressure ≥130/85 mmHg or active antihypertensive pharmacotherapy; fasting plasma triglycerides ≥150 mg/dL (≥1.7 mmol/L) or lipid-lowering medication; high-density lipoprotein cholesterol <40 mg/dL (<1.0 mmol/L) in males and <50 mg/dL (<1.3 mmol/L) in females. Exclusion criteria encompass a historic or current high alcohol consumption (> 30 g/d for men, > 20 g/d for women, ≥ 5 years) (18), viral hepatitis, drug-induced liver disease, Wilson’s disease, autoimmune liver diseases, and other etiologies of hepatic steatosis. Concomitant medication use was systematically recorded at enrollment. Patients on lipid-lowering, hypoglycemic, systemic corticosteroids or known hepatotoxic medication for more than 1 month were excluded.

This cross-sectional study was approved by the Xin Hua Hospital Research Ethics Committee (XHEC-C-2012-023), with written informed consent obtained from each participant. Patients were originally enrolled under NAFLD criteria and retrospectively reclassified using contemporary MASLD diagnostic criteria (EASL-EASD-EASO 2024) (1, 2). All patients fulfilled MASLD criteria without exclusion, because the MASLD definition is broader and inclusive of the original NAFLD phenotype. Supplementary Table S1 displays the baseline information of the cohort.

### 2.2 Hepatic pathological assessment

Liver specimens were collected from each MASLD patient *via* ultrasound-guided percutaneous needle biopsy after obtaining informed consent. Tissues were fixed in 10% neutral-buffered formalin, paraffin-embedded, and sectioned at 5-μm thickness. Hematoxylin & eosin (HE) and Masson’s trichrome staining were sequentially performed for pathological assessment by three pathologists blinded to clinical data. The steatosis, activity, and fibrosis (SAF) scoring method was applied as follows: (1) Steatosis (S0: <5%; S1: 5–33%; S2: 34–66%; S3: >66%); (2) Activity (sum of lobular inflammation [0: no foci; 1: < 2 foci per 200 × field; 2: 2–4 foci per 200 × field; 3: > 4 foci per 200 × field] and hepatocellular ballooning [0: none; 1: few ballooned cells; 2: many cells/prominent ballooning]); and (3) Fibrosis (F0: none; F1: peri-sinusoidal or portal fibrosis; F2: peri-sinusoidal and periportal fibrosis without bridging; F3: bridging fibrosis; F4: cirrhosis) (1, 2, 19).

### 2.3 Anthropometric and biochemical analysis

Anthropometric parameters including weight, waist circumference, body mass index (BMI, calculated as weight in kilograms divided by height in meters squared), and waist-to-height ratio (WHtR, calculated as waist in centimeters divided by height in centimeters) were measured. Biochemical indices were quantified using a LABOSPECT 008 *α* multichannel automatic analyzer (Hitachi, Tokyo, Japan) with commercial kits: total cholesterol (TC) (Cholesterol E/L-Type CHO M), triglyceride (TG) (L-Type Triglyceride M), high-density lipoprotein cholesterol (HDL-C) (L-Type HDL-C), low-density lipoprotein cholesterol (LDL-C) (L-Type LDL-C), alanine aminotransferase (ALT) (L-Type ALT J2), aspartate aminotransferase (AST) (L-Type AST J2), alkaline phosphatase (ALP) (L-Type ALP J2), *γ*-glutamyltransferase (GGT) (L-Type γ-GT J), and total bilirubin (TBIL) (Total Bilirubin E-HA) (all from FUJIFILM Wako Pure Chemical Corporation, Osaka, Japan).

### 2.4 Genotyping of *SIRT1* polymorphism

Peripheral blood mononuclear cells were isolated from whole blood samples (500 μL) by density-gradient centrifugation at 1,500 rpm for 10 min with subsequent buffy-coat separation. Genomic DNA was extracted using the QiAamp DNA Mini Kit (Qiagen, Venlo, Netherlands) and its concentration and purity were assessed by NanoDrop ND-1000 spectrophotometry and 0.8% agarose gel electrophoresis (Thermo Fisher Scientific, Waltham, MA, USA). A custom Ion AmpliSeq panel targeting *SIRT1* SNPs was constructed based on the dbSNP database.^1^ Template DNA underwent emulsion polymerase chain reaction (PCR) on the Ion OneTouch 2 System *per* manufacturer’s protocol (Thermo Fisher Scientific). SNP genotyping was performed by DNA sequencing on an Ion 318 Chip using the Ion PGM System (Thermo Fisher Scientific), followed by data analysis with Auto-user software (Life Technology, Gaithersburg, MD, USA) (20). A total of 15 SNPs were selected for investigation through an integrative strategy combining: (i) dbSNP database screening for variants with minor allele frequency >5% in East Asian populations; (ii) prior candidate-gene and GWAS studies reporting associations with metabolic traits (T2DM, obesity, dyslipidemia); and (iii) functional annotation prioritizing exonic, regulatory (3′-UTR), and tag SNPs (11–17).

### 2.5 Serum lipidomic analysis

Fasting serum samples collected concurrently with liver biopsy were thawed once for lipidomic analysis using an untargeted UPLC-MS/MS approach. Chromatographic separation was achieved on a C8 analytical column (Waters BEH, 2.1 × 100 mm, 1.7 μm) thermostatted at 55 °C, with binary mobile phases containing 10 mM ammonium acetate modifier (phase A: acetonitrile/water, 3:2 v/v; phase B: isopropanol/acetonitrile, 9:1 v/v). A 20-min gradient elution at 0.26 mL/min commenced with 32% B (0–1.5 min), ramped to 85% B by 14 min, further increased to 97% B at 15.5 min (held until 18 min), and returned to initial conditions for re-equilibration (18–20 min).

High-resolution mass spectrometric detection was carried out on a TripleTOF® 5,600 platform (AB SCIEX, Framingham, MA) operating in both ESI polarities under optimized conditions: ion spray voltages of +5.5 and −4.5 kV; interface temperatures of 500 °C (positive) and 600 °C (negative); declustering potential and collision energy fixed at ±100 V and ±10 eV, respectively. To monitor analytical stability, pooled QC samples were randomly distributed throughout the acquisition sequence (*n* = 13). Raw MS data were processed with Analyst® TF 1.6 software. Lipid annotations were performed *via* combined LipidView® and PeakView® analysis against spectral libraries, with subsequent quantification using MultiQuant® 2.0 against isotopically labeled internal standards.

Lipidomic data preprocessing included: (i) batch effect correction using pooled QC samples interspersed every 10 study samples (*n* = 13 QCs total), with intensity drift correction *via* locally estimated scatterplot smoothing (LOESS) regression on QC medians; (ii) missing value imputation using k-nearest neighbors (*k* = 5) for features with <20% missingness, with features exceeding this threshold excluded; (iii) normalization to total ion current (TIC) to account for injection volume variability; and (iv) log₂ transformation to stabilize variance. QC reproducibility was assessed by relative standard deviation (RSD). Following QC-based filtering (relative standard deviation <30% in QC replicates), 239 serum lipid species were retained for final statistical analysis (Supplementary Table S2) (21).

### 2.6 Statistical analysis

The clinical data were expressed as means ± standard deviation (SD). Serum lipidomic profiles were compared between different *SIRT1* SNP genotypes using unpaired Student’s *t*-tests. To account for multiple comparisons in SNP associations with differential serum lipid species, raw *p*-values were adjusted using the Benjamini–Hochberg false discovery rate (FDR) procedure (22). Post-hoc power was calculated using G*Power 3.1 based on observed Cohen’s d values and the actual sample size (23). Effect sizes were interpreted as none (d = 0), negligible (|d| < 0.2), small (0.2 ≤ |d| < 0.5), medium (0.5 ≤ |d| < 0.8), or large (|d| ≥ 0.8). Interaction effects were assessed using ordinal logistic regression with the proportional odds assumption. Likelihood ratio tests (LRT) were employed to compare nested models with and without interaction terms between *SIRT1* SNP genotype and potential effect modifiers (24). Spearman’s correlation was performed to evaluate the associations between serum lipid profile and hepatic histological characteristics. Differences in pathological grading among groups of *SIRT1* were analyzed using the Mann–Whitney U test and chi-square test. SPSS 21.0 (SPSS Inc., Chicago, IL, USA) was used for statistical analysis with a two-sided significance criterion at *p* < 0.05.

## 3 Results

### 3.1 *SIRT1* SNPs associated with serum lipidomics of MASLD patients

Fifteen *SIRT1* SNPs were identified through sequencing, including rs3740053, rs2394443, rs182199697, rs2236318, rs7896005, rs2273773, rs34701705, rs114182972, rs201568954, rs2394445, rs2394446, rs4746720, rs752578, rs182180876, and rs1022764. These variants were localized to distinct genomic regions, comprising three exonic (rs182199697, rs2273773, rs114182972), three intronic (rs2236318, rs7896005, rs34701705), six 3’-UTR (rs201568954, rs2394445, rs2394446, rs4746720, rs752578, rs182180876), two upstream (rs3740053, rs2394443), and one downstream SNP (rs1022764). Comprehensive lipidomic profiling quantified 239 serum lipids across five major classes, including fatty acyls, glycerolipids, glycerophospholipids, sphingolipids, and sterol lipids. Their pathophysiological functions are comprehensively summarized in Supplementary Table S3.

To assess the influence of *SIRT1* polymorphisms on serum lipidomic signatures, we performed an exploratory screening for their associations in Chinese Han patients with MASLD. Seven *SIRT1* SNPs (rs3740053, rs2394443, rs2236318, rs7896005, rs2273773, rs2394445, and rs2394446) demonstrated significant associations with multiple lipid species (Table 1; Supplementary Table S4). Following univariate comparison between genotypes with FDR correction for multiple testing, genotype-dependent alterations were evident across cholesterol esters (CE), ceramides (Cer), free fatty acids (FFA), hexosylceramides (HexCer), lysophospholipids (e.g., lysophosphatidylcholine (LPC), lysophosphatidylcholine plasmalogen (LPCO), lysophosphatidylethanolamine (LPE), lysophosphatidylinositol (LPI)), phospholipids (e.g., phosphatidic acid (PA); phosphatidylcholine (PC), choline plasmalogen (PCO), phosphatidylethanolamine (PE), ethanolamine plasmalogen (PEO), phosphatidylinositol (PI)), sphingomyelin (SM), and triacylglycerols (TAG) (Table 1; Supplementary Table S4).

**Table 1:** Effects of *SIRT1* SNPs on serum lipidomics of patients with metabolic dysfunction-associated steatotic liver disease.

SNPs	Number of SNP-associated serum lipids															
	CE	Cer	FFA	HexCer	LPC	LPCO	LPE	LPI	PA	PC	PCO	PE	PEO	PI	SM	TAG
rs3740053 (A/A:A/G+G/G)	0	0	2	0	2	0	1	0	0	0	0	2	3	0	1	2
rs2394443 (G/G:G/C+C/C)	1	0	0	0	0	0	0	0	0	0	0	0	0	0	0	0
rs2236318 (T/T:T/A+A/A)	1	0	0	1	0	0	0	0	0	0	0	0	0	1	0	2
rs7896005 (A/A:A/G+G/G)	1	0	0	2	2	0	0	0	0	0	0	0	0	0	0	7
rs2273773 (T/T:T/C+C/C)	1	0	0	0	0	0	1	0	1	0	0	0	2	0	0	0
rs2394445 (A/A:A/T+T/T)	1	0	0	0	3	1	0	0	1	1	2	0	2	0	0	7
rs2394446 (A/A:A/T+T/T)	0	3	2	0	1	0	0	1	1	4	0	2	0	0	5	0

CE, cholesterol ester; Cer, ceramide; FFA, free fatty acid; HexCer, hexosaccharide ceramide; LPC, lysophosphatidylcholine; LPCO, lysophosphatidylcholine plasmalogen; LPE, lysophosphatidylethanolamine; LPI, lysophosphatidylinositol; PA, phosphatidic acid; PC, phosphatidylcholine; PCO, choline plasmalogen; PE, phosphatidylethanolamine; PEO, ethanolamine plasmalogen; PI, phosphatidylinositol; SM, sphingomyelin; TAG, triacylglycerol.

### 3.2 *SIRT1* rs2236318, rs7896005 and rs2394445 exhibited dominant effect on TAGs

*SIRT1* rs2236318, rs7896005 and rs2394445, with their effects on 2, 7, and 7 different serum triacylglycerols respectively, dominated the *SIRT1* SNP-lipidomics association (Table 2). Compared to those with the T/A or A/A genotype, MASLD patients carrying the T/T genotype at *SIRT1* rs2236318 demonstrated significantly lower serum levels of TAGs (58:7, 58:8) (Table 2). Similarly, A/A genotypes at *SIRT1* rs7896005 demonstrated significantly lower serum levels of TAGs (56:8, 58:7, 58:8, 58:9, 58:10, 58:11 and 60:12) when compared to those of A/G + G/G genotype (Table 2). In contrast, quantitative analysis showed significantly increased levels of TAGs (48:0, 50:0, 50:1, 52:0, 52:1, 52:2 and 54:1) in the MASLD patients with the A/A phenotype compared to those with the A/T + T/T phenotype at *SIRT1* rs2394445 (Table 2). Both saturated fatty acid (SFA)- and monounsaturated fatty acid (MUFA)-containing TAGs influenced by rs2394445 represented the most abundant species in the serum lipidome, reflecting the primary effect of *SIRT1* polymorphism on circulating lipids.

**Table 2:** *SIRT1* SNPs associate with serum triacylglycerols in patients with metabolic dysfunction-associated steatotic liver disease.

Lipid metabolites	rs2236318			rs7896005			rs2394445		
	T/T (*n* = 23)	T/A+A/A (*n* = 11)	FDR-adjusted *P*	A/A (*n* = 22)	A/G+G/G (*n* = 12)	FDR-adjusted *P*	A/A (*n* = 12)	A/T+T/T (*n* = 22)	FDR-adjusted *P*
TAG 48:0	1.556 ± 1.382	1.663 ± 0.760	0.976	1.606 ± 1.393	1.554 ± 0.807	0.953	2.260 ± 1.533	1.211 ± 0.786	0.036*
TAG 50:0	0.629 ± 0.645	0.630 ± 0.346	0.976	0.651 ± 0.651	0.585 ± 0.361	0.953	0.966 ± 0.744	0.442 ± 0.310	0.034*
TAG 50:1	8.541 ± 5.699	9.938 ± 4.489	0.852	8.842 ± 5.643	9.208 ± 4.898	0.953	11.875 ± 6.063	7.285 ± 4.056	0.036*
TAG 52:0	0.135 ± 0.105	0.144 ± 0.091	0.976	0.138 ± 0.106	0.136 ± 0.089	0.953	0.199 ± 0.132	0.104 ± 0.050	0.034*
TAG 52:1	1.993 ± 1.638	2.133 ± 1.413	0.976	2.063 ± 1.640	1.979 ± 1.434	0.953	3.036 ± 1.877	1.469 ± 0.964	0.034*
TAG 52:2	16.370 ± 7.471	18.716 ± 7.003	0.782	16.682 ± 7.493	17.88 ± 7.199	0.953	21.006 ± 8.544	14.799 ± 5.412	0.036*
TAG 54:1	0.178 ± 0.133	0.206 ± 0.199	0.959	0.183 ± 0.134	0.193 ± 0.194	0.953	0.276 ± 0.199	0.135 ± 0.089	0.034*
TAG 56:8	0.154 ± 0.083	0.207 ± 0.070	0.088	0.148 ± 0.079	0.215 ± 0.072	0.026*	0.162 ± 0.075	0.183 ± 0.093	0.624
TAG 58:7	0.125 ± 0.049	0.184 ± 0.063	0.043*	0.123 ± 0.049	0.182 ± 0.060	0.011*	0.155 ± 0.066	0.138 ± 0.055	0.588
TAG 58:8	0.080 ± 0.035	0.117 ± 0.036	0.043*	0.077 ± 0.034	0.118 ± 0.034	0.011*	0.094 ± 0.042	0.091 ± 0.038	0.898
TAG 58:9	0.178 ± 0.094	0.267 ± 0.113	0.088	0.170 ± 0.088	0.275 ± 0.111	0.011*	0.226 ± 0.114	0.199 ± 0.105	0.619
TAG 58:10	0.071 ± 0.042	0.110 ± 0.066	0.088	0.066 ± 0.034	0.117 ± 0.066	0.011*	0.085 ± 0.048	0.085 ± 0.057	0.984
TAG 58:11	0.016 ± 0.011	0.021 ± 0.013	0.187	0.014 ± 0.009	0.023 ± 0.014	0.040*	0.018 ± 0.013	0.018 ± 0.013	0.984
TAG 60:12	0.010 ± 0.008	0.015 ± 0.007	0.108	0.009 ± 0.006	0.017 ± 0.009	0.011*	0.013 ± 0.011	0.726 ± 0.289	0.495

TAG, triacylglycerol; values are expressed as mean ± SD. ^∗^*p* < 0.05.

Post-hoc power analysis revealed that the study achieved >80% power to detect large effect sizes (Cohen’s d = 0.82–1.12 for *SIRT1* rs2394445-TAGs associations) for SFA- and MUFA-containing TAGs (48:0, 50:0, 50:1, 52:0, 52:1, and 54:1) (Table 3). However, power was insufficient (<30%) for detecting associations with long-chain polyunsaturated TAGs (56:5. 56:6, 56:8, 58:7, 58:8, 58:9, 58:10, 58:11, and 60:12), consistent with their lack of significant genotype-dependent differences in this cohort (Table 3). The rs2394445 genotype explained 16.2 to 23.9% of the variance in serum levels of SFA- and MUFA-containing TAG (*r*^2^ = 0.162–0.239), with the largest proportion of variance explained for TAG 52:1 (Table 3).

**Table 3:** *Post-hoc* power analysis of the association between *SIRT1* rs2394445 and serum triacylglycerols.

TAG species	Cohen’s d	95% CI	*r* ^2^	Variance explained (%)	Achieved power (%)	Effect magnitude
TAG 48:0	0.88	0.21–1.53	0.162	16.2	82.3	Large
TAG 50:0	0.91	0.23–1.57	0.171	17.1	84.5	Large
TAG 50:1	0.89	0.22–1.55	0.165	16.5	83.1	Large
TAG 52:0	0.97	0.28–1.63	0.19	19	88.2	Large
TAG 52:1	1.12	0.42–1.80	0.239	23.9	94.2	Large
TAG 52:2	0.88	0.21–1.53	0.162	16.2	82.3	Large
TAG 54:1	0.9	0.23–1.56	0.168	16.8	83.6	Large
TAG 56:8	−0.25	−0.88 to 0.39	0.015	1.5	12.3	Negligible
TAG 58:7	0.28	−0.37 to 0.91	0.019	1.9	15.8	Small
TAG 58:8	0.08	−0.57 to 0.71	0.002	0.2	5.6	Negligible
TAG 58:9	0.25	−0.40 to 0.89	0.015	1.5	12.8	Small
TAG 58:10	0	−0.65 to 0.65	0	0	5	None
TAG 58:11	0	−0.65 to 0.65	0	0	5	None
TAG 60:12	0.35	−0.30 to 0.99	0.03	3	22.5	Small

TAG, triacylglycerol.

### 3.3 *SIRT1* rs2394445-dependent high-level TAGs correlated to severity of hepatic steatosis

**Figure 1:**
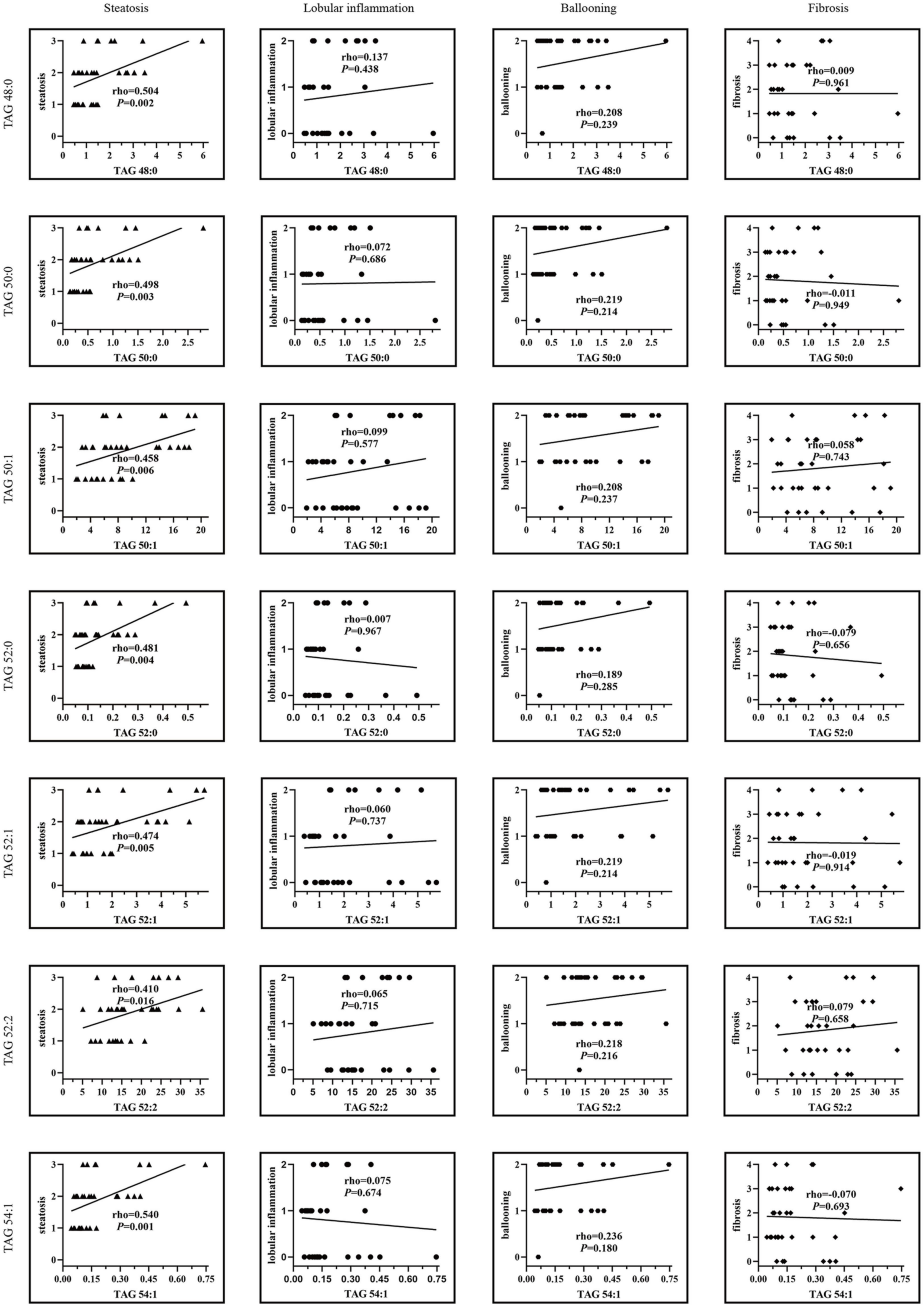
Correlation between pathological characteristics of metabolic dysfunction-associated steatotic liver disease (MASLD) and serum triacylglycerols (TAGs) affected by *SIRT1* rs2394445.

To clarify the potential effect of *SIRT1* rs2394445-dependent lipidomic characteristics on MASLD, we next examined the correlations between TAG species and MASLD-specific pathological characteristics (hepatocyte steatosis, lobular inflammation, ballooning, and liver fibrosis). SFA-containing TAGs (48:0, 50:0, 52:0) and MUFA-containing TAGs (50:1, 52:1, 54:1) rose in parallel with the severity of hepatic steatosis, exhibiting comparable Spearman’s coefficients (rho: 0.367 to 0.540, *p* < 0.033–0.001). (Figure 1). TAG containing double bonds (52:2) correlated with the grade of steatosis in the same pattern (Figure 1). These TAG species accumulated to higher levels in patients carrying the A/A genotype at *SIRT1* rs2394445 than in those with A/T or T/T alleles. Conversely, the remaining MASLD pathological traits showed only weak associations with serum SFA- and MUFA-containing TAGs (Figures 1). Given its impact on the TAG profile, *SIRT1* rs2394445 is associated with hepatic steatosis of MASLD, likely through SFA- and MUFA-containing TAGs. However, the modest sample size limits statistical power for detecting moderate or small associations with histological traits. The absence of significant correlations with inflammation, ballooning, and fibrosis does not exclude the possibility of true biological associations.

### 3.4 A/A genotype at *SIRT1* rs2394445 was associated with severe steatosis

**Figure 2:**
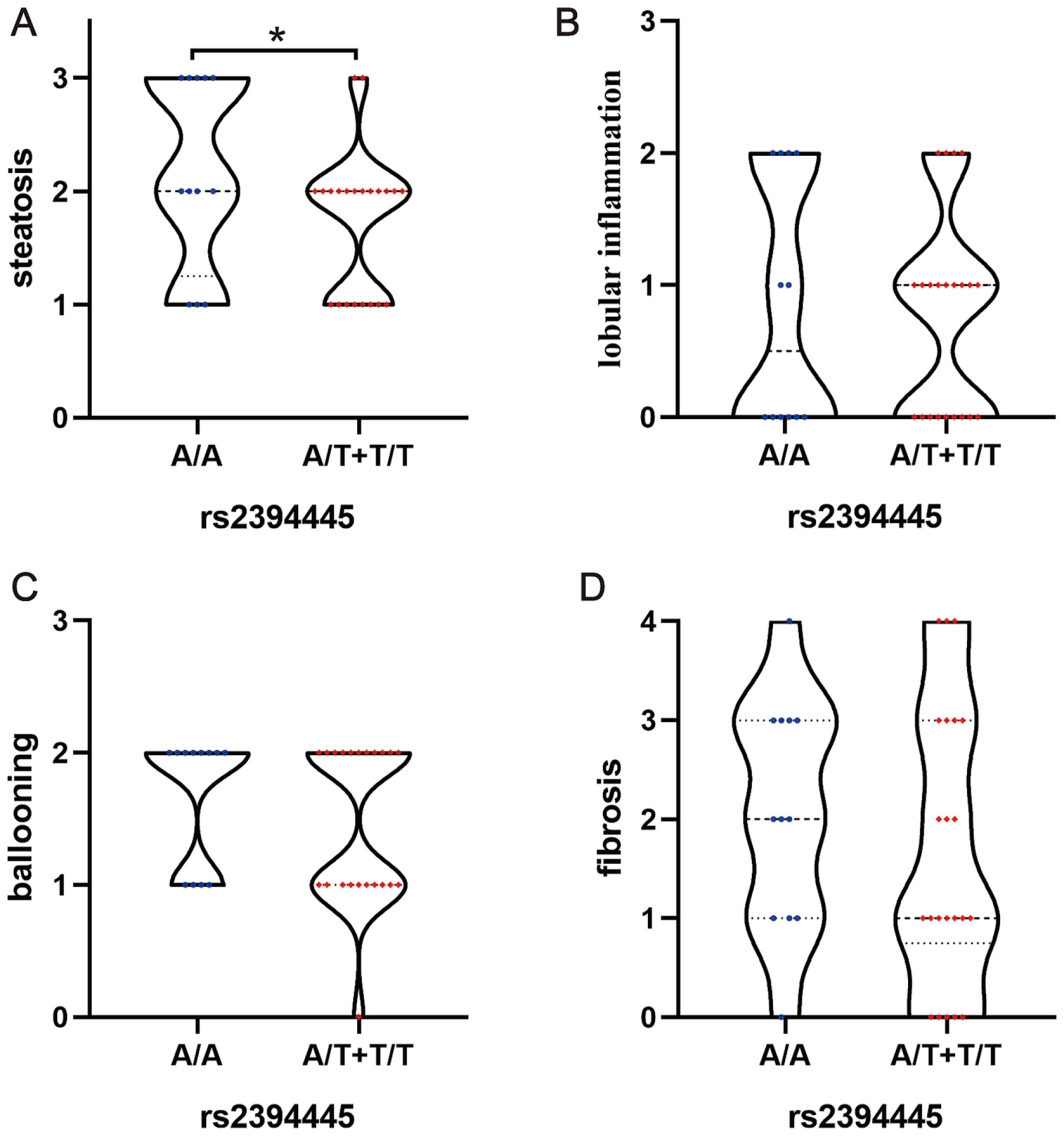
*SIRT1* rs2394445 associated with high-grade hepatic steatosis in MASLD patients. Violin plots indicate the differences in pathologic characteristics of steatosis **(A)**; lobular inflammation **(B)**; ballooning **(C)**; and fibrosis **(D)** between MASLD patients with the A/A or A/T+T/T genotype at *SIRT1* rs2394445. Median and interquartile range are overlaid as dashed lines inside each plot. *, *p* < 0.05.

After stratifying MASLD patients by *SIRT1* rs2394445 genotype, we graded steatosis, lobular inflammation, ballooning, and fibrosis to explore the variant’s histological impact. Steatosis grades 1, 2, and 3 were classified as mild, moderate, and severe fatty liver, respectively. Carriers of the A/A genotype exhibited significantly more advanced steatosis than those with A/T+T/T genotypes (Figures 2, *p* = 0.025), showing a medium effect size (*φ* = 0.385). Beyond steatosis, *SIRT1* rs2394445 showed no significant influence on MASLD-specific lobular inflammation, ballooning, or fibrosis (Figure 2).

Consistently, circulating markers of hepatic inflammation (ALT, AST) did not differ between A/A and A/T+T/T groups (Table 4). Indices reflecting hepatocellular synthetic capacity (ALB) and bile formation/excretion (TBIL, ALP, GGT), the functions typically compromised in fibrosis, were comparable across genotypes (Table 4), aligning with the histological findings. Collectively, the A allele of *SIRT1* rs2394445 is associated with severe hepatic steatosis, an effect closely linked to elevated serum TAG species enriched in saturated and monounsaturated fatty acids.

**Table 4:** Association of *SIRT1* rs2394445 and anthropometric, biochemical indices.

Clinical parameter	Genotype grouping		*p*-value
	A/A	A/T + T/T	
Gender	M: 4 (33.3%)	M: 15 (68.2%)	0.058
	F: 8 (66.7%)	F: 7 (31.8%)	
Age (years)	40.08 ± 16.05	41.55 ± 14.45	0.788
Weight (kg)	69.83 ± 10.29	78.34 ± 7.71	0.010*
Height (m)	1.641 ± 0.070	1.693 ± 0.078	0.063
Waist (cm)	88.83 ± 7.15	91.55 ± 6.25	0.259
BMI (kg/m2)	25.98 ± 3.81	27.39 ± 2.65	0.214
WHtR	0.44 ± 0.01	0.39 ± 0.08	0.978
ALB (mg/dl)	42.45 ± 5.58	40.77 ± 8.51	0.469
ALT (U/L)	59.36 ± 38.01	76.53 ± 63.99	0.403
AST (U/L)	47.03 ± 21.15	54.78 ± 40.54	0.469
ALP (U/L)	104.69 ± 58.38	94.89 ± 43.48	0.582
GGT (U/L)	105.94 ± 175.69	135.09 ± 343.87	0.786
TBIL (μmol/L)	20.68 ± 26.18	13.87 ± 3.68	0.389
TC (mmol/L)	4.76 ± 0.81	4.66 ± 0.55	0.669
TG (mmol/L)	1.88 ± 0.88	1.61 ± 0.59	0.350
HDL-C (mmol/L)	1.14 ± 0.35	1.17 ± 0.25	0.800
LDL-C (mmol/L)	2.80 ± 0.74	2.64 ± 0.43	0.435

WHtR, waist-to-height ratio; ALB, albumin; ALT, alanine aminotransferase; AST, aspartate aminotransferase; ALP, alkaline phosphatase; GGT, gamma-glutamyl transpeptidase; TBIL, total bilirubin; TC, total cholesterol; TG, total triglyceride; HDL-C, high-density lipoprotein cholesterol; LDL-C, low-density lipoprotein cholesterol.

### 3.5 *SIRT1* rs2394445 effected on hepatic steatosis independent of total circulating lipids and obesity

To determine whether *SIRT1* rs2394445 influences hepatic steatosis *via* total circulating lipids, we compared fasting serum levels of TC, TG, LDL-C, and HDL-C. No statistical differences were observed between A/A carriers and A/T+T/T carriers (Table 4). Despite the height-related difference in body weight, this variant likewise displayed insignificant effect on both generalized obesity (BMI) and abdominal obesity (WHtR) (Table 4). By ordinal logistic regression, no significant effect modification was observed for any interaction term (Supplementary Table S5), suggesting that the effect of rs2394445 on steatosis severity was independent of gender, age and other clinical characteristics. These results suggest that the impact of *SIRT1* rs2394445 on lipid distribution is exerted predominantly within the liver.

## 4 Discussion

This exploratory pilot study integrated *SIRT1* genotyping, untargeted serum lipidomics, and biopsy-based histopathological assessment to investigate genotype-lipid-phenotype relationships in Chinese Han patients with MASLD. Our principal finding exhibits that the A/A genotype at *SIRT1* rs2394445 is associated with elevated circulating triacylglycerols enriched in saturated and monounsaturated fatty acids (SFA-TAGs and MUFA-TAGs), which in turn correlate with high-grade hepatic steatosis. This association appears independent of total circulating lipids and BMI-defined obesity, suggesting a liver-intrinsic metabolic mechanism.

SIRT1 has been recognized to regulate the metabolism of triacylglycerol species through distinctive mechanisms, including deacetylation of substrate-specific enzymes and selective modulation of transcription factors, *etc*. It inactivates SREBP-1c *via* deacetylation, thereby inhibiting the nuclear translocation of SREBP-1c, down-regulating the transcription of lipogenic genes such as SCD1 and FASN, and reducing both SFA synthesis and their desaturation to monounsaturated fatty acids (25). In addition, SIRT1 suppresses GPAM and AGPAT2, preventing SFA from esterifying with the glycerol backbone to form triacylglycerol precursors. By inhibiting DGAT2, SIRT1 selectively blocks the esterification of SFA/MUFA at key nodes of the triacylglycerol biosynthetic pathway (26). Furthermore, SIRT1 promotes AMPK phosphorylation and activation through deacetylation of LKB1, then redirecting metabolic flux from lipogenesis toward fatty acid oxidation and indirectly curtailing the incorporation of SFA/MUFA into triacylglycerol pools (27).

In the present study, *SIRT1* variants (e.g., rs3740053, rs2394445) were associated with differential abundance of multiple lipid classes, with triacylglycerols being the most affected. Notably, rs2394445 A allele carriers showed elevated levels of saturated and monounsaturated TAG species (48:0, 50:0, 50:1, 52:0, 52:1, and 54:1) compared to T allele carriers, whereas rs2236318 and rs7896005 primarily influenced polyunsaturated TAGs. Since the TAG species modulated by rs2394445 represent the most abundant serum lipid constituents, this variant exerts a quantitatively dominant effect on the circulating lipidome.

Clinical evidence has shed light on the close association between SFA/MUFA species and metabolic disorders (28–31). Diet-induced weight loss reduces the levels of triacylglycerols containing saturated short-chain fatty acids that correlate with enhanced insulin sensitivity (28). In contrast, serum triacylglycerols enriched in saturated and monounsaturated fatty acids, such as palmitic acid (16:0), palmitoleic acid (16:1 n-7), and oleic acid (18:1 n-9), are positively associated with HOMA-IR (29, 30). Consistent with these serum lipidomic patterns, insulin resistance-driven MASLD patients exhibit depletion of n-6 and n-3 long-chain polyunsaturated fatty acids in hepatic triacylglycerols, as evidenced by decreased 20:4 n-6/18:2 n-6 and (20:5 n-3 + 22:6 n-3)/18:3 n-3 ratios (31).

Our experiments demonstrated that triacylglycerols containing saturated fatty acids (SFA-TAGs, such as TAG 48:0, TAG 50:0, and TAG 52:0) and those containing monounsaturated fatty acids (MUFA-TAGs, such as TAG 50:1, TAG 52:1, and TAG 54:1) showed significant positive correlations with the severity of biopsy-proven steatosis. Most of these TAGs shared comparable Spearman rank correlation coefficients. Given that rs2394445 A allele carriers display elevated saturated and monounsaturated TAG levels compared to those with T allele, this SNP is proposed to associate with hepatic steatosis.

Indeed, our findings in MASLD patients demonstrated that high levels of SFA-TAGs and MUFA-TAGs, together with severe hepatic steatosis, characterized patients with A/A genotype at *SIRT1* rs2394445. The close correlation between *SIRT1* rs2394445 genotype and steatosis grading suggests a potential TAG-related association in MASLD pathophysiology. In brief, there was a significantly higher grade of hepatic steatosis in patients with the A/A versus A/T+T/T genotype at *SIRT1* rs2394445. But neither *SIRT1* rs2394445 nor these TAG species correlated with other pathological features, including lobular inflammation, ballooning, or liver fibrosis. Salomone *et al* recently reported that the *SIRT5* rs12216101 T>G variant confers liver damage, mitochondrial dysfunction, and oxidative stress in European MASLD patients (32). This discrepancy highlights divergent effects of *SIRT* family SNPs on the MASLD pathological phenotypes.

The discordance between severe steatosis and other pathological characteristics in rs2394445 A/A carriers warrants mechanistic consideration, and larger-scale clinical trials over the long term are necessary for verification. SIRT1 is a potent activator of the FOXO3a-mediated antioxidant defense program, including SOD2 and catalase expression (33). Enhanced SIRT1 activity (or genotype-dependent compensatory upregulation) may preserve hepatocellular redox homeostasis despite lipid overload, thereby limiting necroinflammatory injury and aminotransferase release. Additionally, SIRT1-mediated deacetylation of NF-κB p65 dampens pro-inflammatory signaling (34), potentially explaining the absence of lobular inflammation despite high-grade steatosis. This “metabolically benign” steatosis phenotype, characterized by lipid accumulation without parallel inflammatory injury, has been described in PNPLA3 rs738409 homozygotes, who frequently exhibit high steatosis with relatively preserved ALT levels (6). The hepatoprotective and regenerative capacity of hepatocytes, maintained through SIRT1-PGC-1α mitochondrial biogenesis (35), may further buffer against cell death and enzyme leakage in this cohort. Taken together, robust anti-peroxidation, repair, and regenerative activities of hepatocytes may account for the discordance among these pathological indices (36).

Apart from the lipidomic abnormalities, hepatic steatosis can be driven by a global rise in circulating lipids (e.g., hypertriglyceridaemia, hypercholesterolaemia, elevated LDL-C) (4, 37). In our experiments, fasting serum TC, TG, LDL-C and HDL-C did not differ between *SIRT1* rs2394445 A/A carriers and A/T+T/T carriers. This evidence underscores that the variant’s effect on hepatic steatosis may be linked to a shift in TAG species, specifically an enrichment of SFA- and MUFA-containing TAGs, rather than to a quantitative increase in total circulating lipids. To determine whether the association between *SIRT1* rs2394445 and hepatic steatosis is secondary to adiposity (38, 39), we further compared BMI and WHtR across genotypes and obtained no significant differences. Thus, *SIRT1* rs2394445 may correlate with severe hepatic steatosis through a TAG-centric, liver-intrinsic mechanism independent of both generalized and central obesity.

Our study has several limitations that warrant consideration. Given the exploratory nature with modest sample size, this investigation provides adequate statistical power for detecting large effects, as exemplified by the rs2394445-TAG-steatosis associations. But it is inherently limited to detect medium or small effects, and negative findings should therefore be interpreted as inconclusive to avoid Type II error. Additionally, the cross-sectional design precludes causal inference. As rs2394445 is situated within the 3′-UTR region of *SIRT1*, direct effects on protein conformation are not expected (40). One might speculate that its influence on the triacylglycerol profile could potentially involve regulation of protein expression levels. However, this mechanism remains to be fully explored, as direct evidence linking this SNP to protein expression, splicing, or miRNA binding, has yet to be comprehensively established. Furthermore, the untargeted UPLC-MS/MS approach used for lipidomic analysis provides information on unsaturated bond counts, though precise determination of their exact locations presents certain technical limitations (41).

## 5 Conclusion

*SIRT1* polymorphisms represent an important genetic determinant of serum lipidomic signatures in MASLD, particularly regarding TAG composition. The A/A genotype at rs2394445 is predominantly associated with elevated SFA- and MUFA-enriched TAG species, which correlate with high-grade steatosis. *SIRT1* rs2394445, therefore, may be associated with severe hepatic steatosis in MASLD through its modulatory influence on TAG profiles. This effect is independent of both circulating lipid levels and obesity. However, independent validation is warranted for this exploratory study before any clinical inferences can be made.

## Data Availability

The original contributions presented in the study are publicly available. This data can be found here: 10.6084/m9.figshare.32908523.

## Footnotes

1
https://www.ncbi.nlm.nih.gov/snp/

## Supplementary material

SUPPLEMENTARY TABLE S4 Serum lipid components associated with SIRT1 SNPs in metabolic dysfunction-associated steatotic liver disease.
