# Supplementary material for: SIRT1 rs2394445 associates with serum triacylglycerol lipidomic profile in metabolic dysfunction-associated steatotic liver disease
Source: Front Nutr. 2026 Jul 22;13:1835298. doi: 10.3389/fnut.2026.1835298 (PMC13438381; doi:10.3389/fnut.2026.1835298)
Supplement: Supplementary file 1 [file Supplementary_file_1.DOCX]

**Supplementary Table S1. Baseline information of cohort**

| **Variables** | **Cohort** |
| --- | --- |
| **Demographic characteristics** |  |
| Age (years) | 41.03±14.809 |
| Gender |  |
| Male (n, %) | 19 (55.9%) |
| Female (n, %) | 15 (44.1%) |
| **Anthropometric indices** |  |
| Weight (kg) | 75.34±9.491 |
| Height (m) | 1.674±0.078 |
| Waist (cm) | 90.58±6.61 |
| BMI (kg/m2) | 26.897±3.127 |
| WHtR | 0.542±0.040 |
| **Liver function** |  |
| ALB (mg/dl) | 41.36±7.553 |
| ALT (U/L) | 70.47±56.186 |
| AST (U/L) | 52.04±34.776 |
| ALP (U/L) | 98.35±48.598 |
| GGT (U/L) | 124.80±292.812 |
| TBIL (μmol/L) | 16.27±15.746 |
| **Blood lipid profiles** |  |
| TC (mmol/L) | 4.70±0.642 |
| TG (mmol/L) | 1.71±0.704 |
| HDL-C (mmol/L) | 1.16±0.283 |
| LDL-C (mmol/L) | 2.70±0.557 |
| **Glucose metabolism** |  |
| FBG (mmol/L) | 5.62±1.370 |
| Hemoglobin A1c (%) | 5.99±0.977 |
| **Medical history (n, %)** |  |
| Hypertension | 6 (17.6%) |
| Diabetes mellitus | 4 (11.8%) |
| Dyslipidemia | 18 (52.9%) |
| Long-term medication use^a^ |  |
| Long-term antihypertensive drugs | 4 (11.8%) |
| Long-term hypoglycemic drugs | 2 (5.9%) |
| Long-term lipid-lowering drugs | 0 (0%) |
| **Alcohol consumption** |  |
| Alcohol drinking history (n, %) | 4 (11.8%) |
| Drinking duration (years)^b^ | 17.75 |
| Average daily alcohol intake (g/d)^b^ | 35 |
| **Pathology (n, %)** |  |
| Steatosis |  |
| 0 | 0 (0%) |
| 1 | 11 (32.3%) |
| 2 | 16 (47.1%) |
| 3 | 7 (20.6%) |
| Lobular inflammation |  |
| 0 | 15 (44.1%) |
| 1 | 11 (32.3%) |
| 2 | 8 (23.5%) |
| 3 | 0 (0%) |
| Ballooning |  |
| 0 | 1 (2.9%) |
| 1 | 14 (41.2%) |
| 2 | 19 (55.9%) |
| Fibrosis |  |
| 0 | 6 (17.6%) |
| 1 | 10 (29.4%) |
| 2 | 6 (17.6%) |
| 3 | 8 (23.5%) |
| 4 | 4 (11.8%) |

WHtR: waist-to-height ratio; ALB: albumin; ALT: alanine aminotransferase;

AST: aspartate aminotransferase; ALP: alkaline phosphatase;

GGT: gamma-glutamyl transpeptidase; TBIL: total bilirubin;

TC: total cholesterol; TG: total triglyceride; HDL-C: high-density lipoprotein cholesterol;

LDL-C: low-density lipoprotein cholesterol; FBG: Fasting blood glucose;

^a^Regular medication for more than one month.

^b^Only calculated in participants with alcohol drinking history.

**Supplementary Table S2. Serum lipidomics of cohort**

| **CE** | **Cer** | **DAG** | **HexCer** | **FFA** | **LPC** | **LPCO** | **LPE** | **LPEO** | **LPI** | **PA** | **PC** | | **PCO** | **PE** | **PEO** | **PI** | **SM** | **TAG** | | |
| --- | --- | --- | --- | --- | --- | --- | --- | --- | --- | --- | --- | --- | --- | --- | --- | --- | --- | --- | --- | --- |
| CE 18:1 | Cer 40:1;2 | DAG 34:1 | Hex2Cer 34:1;2 | FFA 12:0 | LPC 14:0 | LPC O-16:0 | LPE 18:0 | LPE O-16:1 | LPI 18:0 | PA 34:2 | PC 30:0 | PC 36:5 | PC O-32:0 | PE 34:0 | PE O-34:2 | PI 32:1 | SM 32:1;2 | TAG 40:0 | TAG 50:4 | TAG 56:2 |
| CE 18:2 | Cer 40:2;2 | DAG 34:2 | HexCer 34:1;2 | FFA 14:0 | LPC 16:0 | LPC O-16:1 | LPE 18:1 | LPE O-18:1 | LPI 20:4 |  | PC 32:0 | PC 36:6 | PC O-34:0 | PE 34:1 | PE O-34:3 | PI 34:1 | SM 32:2;2 | TAG 40:1 | TAG 50:5 | TAG 56:3 |
| CE 18:3 | Cer 41:1;2 | DAG 36:2 | HexCer 40:1;2 | FFA 14:1 | LPC 16:1 | LPC O-18:1 | LPE 20:4 |  |  |  | PC 32:1 | PC 38:1 | PC O-34:1 | PE 34:2 | PE O-36:3 | PI 34:2 | SM 34:0;2 | TAG 42:0 | TAG 52:0 | TAG 56:4 |
| CE 20:4 | Cer 42:1;2 | DAG 36:3 |  | FFA 16:0 | LPC 17:0 |  | LPE 22:6 |  |  |  | PC 32:2 | PC 38:2 | PC O-34:2 | PE 36:1 | PE O-36:5 | PI 36:1 | SM 34:0;3 | TAG 42:1 | TAG 52:1 | TAG 56:5 |
| CE 20:5 | Cer 42:2;2 | DAG 36:4 |  | FFA 16:1 | LPC 18:0 |  |  |  |  |  | PC 34:1 | PC 38:3 | PC O-34:3 | PE 36:2 | PE O-36:6 | PI 36:2 | SM 34:1;2 | TAG 42:2 | TAG 52:2 | TAG 56:6 |
| CE 22:6 |  |  |  | FFA 18:0 | LPC 18:1 |  |  |  |  |  | PC 34:2 | PC 38:4 | PC O-36:2 | PE 36:3 | PE O-38:5 | PI 36:3 | SM 34:1;3 | TAG 44:0 | TAG 52:3 | TAG 56:7 |
|  |  |  |  | FFA 18:1 | LPC 18:2 |  |  |  |  |  | PC 34:3 | PC 38:5 | PC O-36:3 | PE 36:4 | PE O-38:6 | PI 36:4 | SM 34:2;2 | TAG 44:1 | TAG 52:4 | TAG 56:8 |
|  |  |  |  | FFA 18:2 | LPC 18:3 |  |  |  |  |  | PC 34:4 | PC 38:6 | PC O-36:4 | PE 38:4 | PE O-38:7 | PI 38:3 | SM 34:2;3 | TAG 44:2 | TAG 52:5 | TAG 56:9 |
|  |  |  |  | FFA 18:3 | LPC 20:0 |  |  |  |  |  | PC 34:5 | PC 38:7 | PC O-36:5 | PE 38:5 | PE O-40:5 | PI 38:4 | SM 36:0;2 | TAG 46:0 | TAG 52:6 | TAG 58:1 |
|  |  |  |  | FFA 20:0 | LPC 20:1 |  |  |  |  |  | PC 36:0 | PC 40:4 | PC O-38:4 | PE 38:6 | PE O-40:7 | PI 40:4 | SM 36:1;2 | TAG 46:1 | TAG 52:7 | TAG 58:10 |
|  |  |  |  | FFA 20:1 | LPC 20:2 |  |  |  |  |  | PC 36:1 | PC 40:5 | PC O-38:5 | PE 40:5 | PE O-40:8 | PI 40:5 | SM 36:2;2 | TAG 46:2 | TAG 54:0 | TAG 58:11 |
|  |  |  |  | FFA 20:2 | LPC 20:3 |  |  |  |  |  | PC 36:2 | PC 40:6 | PC O-38:6 | PE 40:6 | PE O-42:7 | PI 40:6 | SM 36:3;2 | TAG 46:3 | TAG 54:1 | TAG 58:3 |
|  |  |  |  | FFA 20:3 | LPC 20:4 |  |  |  |  |  | PC 36:3 | PC 40:7 | PC O-38:7 |  |  |  | SM 38:1;2 | TAG 48:0 | TAG 54:2 | TAG 58:4 |
|  |  |  |  | FFA 20:4 | LPC 20:5 |  |  |  |  |  | PC 36:4 | PC 40:8 | PC O-40:4 |  |  |  | SM 38:2;2 | TAG 48:1 | TAG 54:3 | TAG 58:5 |
|  |  |  |  | FFA 20:5 | LPC 22:6 |  |  |  |  |  |  |  | PC O-40:5 |  |  |  | SM 40:1;2 | TAG 48:2 | TAG 54:4 | TAG 58:6 |
|  |  |  |  | FFA 22:0 | LPC 24:0 |  |  |  |  |  |  |  | PC O-40:6 |  |  |  | SM 40:2;2 | TAG 48:3 | TAG 54:5 | TAG 58:7 |
|  |  |  |  | FFA 22:1 |  |  |  |  |  |  |  |  | PC O-42:4 |  |  |  | SM 40:3;2 | TAG 48:4 | TAG 54:6 | TAG 58:8 |
|  |  |  |  | FFA 22:2 |  |  |  |  |  |  |  |  | PC O-42:5 |  |  |  | SM 42:1;2 | TAG 50:0 | TAG 54:7 | TAG 58:9 |
|  |  |  |  | FFA 22:4 |  |  |  |  |  |  |  |  | PC O-42:6 |  |  |  | SM 42:1;3 | TAG 50:1 | TAG 54:8 | TAG 60:12 |
|  |  |  |  | FFA 22:5 |  |  |  |  |  |  |  |  | PC O-44:5 |  |  |  | SM 42:2;2 | TAG 50:2 | TAG 56:0 | TAG 60:2 |
|  |  |  |  | FFA 22:6 |  |  |  |  |  |  |  |  | PC O-44:6 |  |  |  | SM 42:3;2 | TAG 50:3 | TAG 56:1 |  |
|  |  |  |  | FFA 24:0 |  |  |  |  |  |  |  |  |  |  |  |  | SM 44:2;2 |  |  |  |
|  |  |  |  |  |  |  |  |  |  |  |  |  |  |  |  |  | SM 44:3;2 |  |  |  |

CE: cholesterol ester; Cer: ceramide; DAG: diacylglycerol; HexCer: hexosaccharide ceramide; FFA: free fatty acid; LPC: lysophosphatidylcholine; LPCO: lysophosphatidylcholine

plasmalogen; LPE: lysophosphatidylethanolamine; LPEO: lysophosphatidylethanolamine plasmalogen; LPI: lysophosphatidylinositol; PA: phosphatidic acid; PC: phosphatidylcholine; PCO: choline plasmalogen; PE: phosphatidylethanolamine; PEO: ethanolamine plasmalogen; PI: phosphatidylinositol; SM: sphingomyelin; TAG: triacylglycerol

## **Supplementary Table S3. Pathophysiological functions of lipid classes**

| **Lipid Class** | **Primary Pathophysiological Functions** |
| --- | --- |
| CE (Cholesterol Ester) | ① Storage and transport form of cholesterol, accumulates in lipid droplets and lipoproteins(1); ② Excess CE causes cholesterol overload in hepatocytes, disrupting mitochondrial and ER membrane stability, promoting steatohepatitis and fibrosis(1, 2); ③ Participates in atherosclerotic plaque formation(3) |
| Cer (Ceramide) | ① Key mediator of saturated FFA-induced lipotoxicity, induces insulin resistance via JNK, PKCθ activation(1, 4); ② Promotes mitochondrial dysfunction, ROS production, and ER stress(1, 4); ③ Activates inflammasome (NLRP3), driving macrophage activation and hepatic fibrosis(1, 4); ④ Inhibits Akt/PKB signaling, promoting hepatocyte apoptosis(4) |
| DAG (Diacylglycerol) | ① Activates PKCε, leading to IRS-1 serine phosphorylation-a core molecular mechanism of hepatic insulin resistance(1, 5); ② Promotes mitochondrial dysfunction and oxidative stress(1); ③ As an intermediate in TG synthesis, its accumulation reflects excessive lipogenic pathway activity(1, 2); ④ Closely associated with MASLD progression to MASH(1, 2) |
| HexCer (Hexosylceramide) | ① Terminal product of sphingolipid metabolism toward glycosphingolipids, reflecting ceramide metabolic reprogramming(4); ② Significantly elevated in hepatocellular carcinoma, promoting tumor cell proliferation and membrane synthesis(4); ③ Participates in cell signal transduction and immune regulation(4); ④ Potential biomarker in chronic hepatitis and cirrhosis(2) |
| FFA (Free Fatty Acid) | ① Major source of lipid overload, enters hepatocytes in large quantities via CD36/FATP transporters(1, 6); ② Saturated FFAs (e.g., palmitate) directly induce lipotoxicity, activating TLR4/NF-κB inflammatory pathways(1, 6); ③ Promotes ER stress and unfolded protein response (UPR)(1); ④ Serves as substrate for synthesis of toxic lipids (Cer, DAG)(1, 4) |
| LPC (Lysophosphatidylcholine) | ① Primarily produced from PC via PLA₂ hydrolysis, a pro-inflammatory lipid component in oxidized LDL(3); ② Induces endothelial cell activation, monocyte chemotaxis, and atherosclerosis(3); ③ High concentrations disrupt mitochondrial integrity, promoting cytochrome C release and apoptosis(3); ④ Upregulates cholesterol synthesis genes and downregulates fatty acid oxidation genes in liver(3) |
| LPCO (LPC Plasmalogen) | ① Plasmalogens possess antioxidant properties, protecting cell membranes from lipid peroxidation damage(7); ② LPCO level changes reflect phospholipid remodeling and oxidative stress status(7); ③ Associated with PNPLA3 polymorphism in MASLD, participating in inflammatory regulation(8); ④ May affect hepatocyte function by modulating membrane fluidity and signal transduction(7) |
| LPE (Lysophosphatidylethanolamine) | ① Possesses anti-inflammatory and mitochondrial protective effects; decreased serum LPE correlates with MASH progression(9); ② Promotes hepatocyte lipid droplet formation, participating in steatosis(9); ③ Over-degraded via Pnpla6/7-mediated pathways in MASH, leading to protective depletion(9); ④ Specific species (e.g., LPE 18:1) show differential changes in disease(9) |
| LPEO (LPE Plasmalogen) | ① Plasmalogens have antioxidant and membrane-stabilizing functions, protecting hepatocytes from oxidative damage(7); ② Decreased in neurodegenerative and metabolic diseases(7); ③ Participates in membrane lipid remodeling and signaling molecule generation(7); ④ May exert protective effects by modulating inflammatory responses and mitochondrial function(7) |
| LPI (Lysophosphatidylinositol) | ① Precursor of phosphatidylinositol signaling pathway, involved in cell proliferation, survival, and metabolic regulation(5); ② Lysophosphatidyl form may regulate membrane permeability and ion channel function(5); ③ Less studied in liver disease, potentially involved in inflammation and fibrosis processes(2); ④ Reflects membrane remodeling status as part of phospholipid metabolic network(2, 5) |
| PA (Phosphatidic Acid) | ① Key intermediate in TG synthesis (GPAT→LPA→PA→DAG→TAG); accumulation reflects lipogenic pathway overload(1, 5); ② As a lipid signaling molecule, PA can activate mTORC2 and inhibit insulin signaling (e.g., di16:0-PA)(5); ③ Regulates nuclear lipid signaling and gene expression(5); ④ Directly associated with hepatic insulin resistance and steatosis(1, 5) |
| PC (Phosphatidylcholine) | ① Major phospholipid component of cell membranes (~40–50%), maintaining membrane structure and fluidity(1, 2); ② Participates in VLDL assembly and secretion; PC deficiency impairs hepatic lipid export(1, 2); ③ Participates in fatty acid metabolism regulation via enzymes such as LPCAT3(1, 8); ④ Essential phospholipids (e.g., polyenylphosphatidylcholine) can improve hepatic steatosis(1) |
| PCO (PC Plasmalogen) | ① Plasmalogens possess strong antioxidant capacity, scavenging free radicals to protect cell membranes(7); ② Synthesized in peroxisomes; their levels reflect peroxisomal function(7); ③ Enhances lipolysis by inhibiting p75NTR, alleviating steatosis(7); ④ Improves cognitive dysfunction and neurodegenerative disorders(7) |
| PE (Phosphatidylethanolamine) | ① Major phospholipid of inner mitochondrial membrane, essential for electron transport chain and oxidative phosphorylation(1); ② Participates in autophagy and mitophagy regulation(1); ③ PE/PC ratio imbalance affects membrane curvature and organelle function(1); ④ May decrease in steatosis, affecting mitochondrial integrity(1, 2) |
| PEO (PE Plasmalogen) | ① Ethanolamine plasmalogen (PlsEtn) enriched in brain and liver, possessing antioxidant and anti-inflammatory properties(7); ② Peroxisomal dysfunction reduces PlsEtn synthesis, exacerbating steatosis and cognitive impairment(7); ③ Regulates neuroprotection and lipid metabolism via TrkA/p75NTR balance(7); ④ PlsEtn supplementation improves steatohepatitis and memory impairment(7) |
| PI (Phosphatidylinositol) | ① Core component of phosphatidylinositol signaling system; PIP₂ hydrolysis produces IP₃ and DAG, regulating Ca²⁺ signaling(5); ② Participates in insulin signal transduction and GLUT4 translocation(5); ③ Specific PI species decrease in cirrhosis(2); ④ Participates in cytoskeletal reorganization and membrane trafficking(5) |
| SM (Sphingomyelin) | ① Conversion of ceramide to SM can reduce pro-apoptotic effects of Cer, possessing cytoprotective effects(4); ② As a membrane structural lipid, enriched in lipoproteins (e.g., LDL)(4); ③ SM hydrolysis to generate Cer is an important source of elevated Cer in MASLD (acid sphingomyelinase upregulation)(4); ④ Participates in cholesterol transport and membrane microdomain (lipid raft) formation(4) |
| TAG (Triacylglycerol) | ① Major storage lipid form in liver, sequestering excess fatty acids in lipid droplets, initially protective(1, 6); ② TAG itself is relatively inert, but its synthesis produces toxic intermediates (DAG, PA, Cer)(1, 6); ③ MASH patients show increased SFA and MUFA, decreased PUFA in TAG(2, 10); ④ TAG/VLDL secretion impairment exacerbates hepatic lipid deposition(1, 2) |

**Reference**

1.	Loomba R, Friedman SL, Shulman GI. Mechanisms and disease consequences of nonalcoholic fatty liver disease. Cell. 2021;184(10):2537-64.

2.	Puri P, Baillie RA, Wiest MM, Mirshahi F, Choudhury J, Cheung O, et al. A lipidomic analysis of nonalcoholic fatty liver disease. Hepatology. 2007;46(4):1081-90.

3.	Law SH, Chan ML, Marathe GK, Parveen F, Chen CH, Ke LY. An Updated Review of Lysophosphatidylcholine Metabolism in Human Diseases. Int J Mol Sci. 2019;20(5).

4.	Summers SA. Could Ceramides Become the New Cholesterol? Cell Metab. 2018;27(2):276-80.

5.	Samuel VT, Shulman GI. The pathogenesis of insulin resistance: integrating signaling pathways and substrate flux. J Clin Invest. 2016;126(1):12-22.

6.	Kuchay MS, Choudhary NS, Ramos-Molina B. Pathophysiological underpinnings of metabolic dysfunction-associated steatotic liver disease. American Journal of Physiology-Cell Physiology. 2025;328(5):C1637-C66.

7.	Liu Y, Cong P, Zhang T, Wang R, Wang X, Liu J, et al. Plasmalogen attenuates the development of hepatic steatosis and cognitive deficit through mechanism involving p75NTR inhibition. Redox Biol. 2021;43:102002.

8.	Mancina RM, Valenti L, Romeo S. Human genetics of steatotic liver disease: insights into insulin resistance and lipid metabolism. Nat Metab. 2025;7(11):2199-211.

9.	Inoue N, Ho HJ, SG BG, Eguchi M, Masamura-Takeuchi M, Chiba H, et al. Lysophosphatidylethanolamine Degradation Associated with Upregulation of Pnpla6/7 in a Murine Model of Metabolic Dysfunction-Associated Steatohepatitis. Int J Mol Sci. 2026;27(4).

10.	Orešič M, Hyötyläinen T, Kotronen A, Gopalacharyulu P, Nygren H, Arola J, et al. Prediction of non-alcoholic fatty-liver disease and liver fat content by serum molecular lipids. Diabetologia. 2013;56(10):2266-74.

|  | β | SE | z | *p* (Wald) | OR | 95% CI | LRT χ² | df | *p* (LRT) | Interaction |
| --- | --- | --- | --- | --- | --- | --- | --- | --- | --- | --- |
| rs2394445 × Gender | -0.798 | 1.599 | -0.499 | 0.618 | 0.450 | (0.02, 10.35) | 0.250 | 1 | 0.617 | No |
| rs2394445 × Age | 0.005 | 0.047 | 0.113 | 0.910 | 1.005 | (0.92, 1.10) | 0.013 | 1 | 0.910 | No |
| rs2394445 × TG | 0.322 | 0.931 | 0.346 | 0.730 | 1.380 | (0.22, 8.56) | 0.120 | 1 | 0.730 | No |
| rs2394445 × TC | 0.563 | 0.717 | 0.785 | 0.432 | 1.755 | (0.43, 7.15) | 0.628 | 1 | 0.428 | No |
| rs2394445 × LDL-C | -0.483 | 0.690 | -0.701 | 0.484 | 0.617 | (0.16, 2.38) | 0.493 | 1 | 0.483 | No |
| rs2394445 × HDL-C | 0.712 | 0.701 | 1.015 | 0.310 | 2.037 | (0.52, 8.05) | 1.060 | 1 | 0.303 | No |
| rs2394445 × BMI | 0.095 | 0.225 | 0.424 | 0.672 | 1.100 | (0.71, 1.71) | 0.180 | 1 | 0.672 | No |
| rs2394445 × WHtR | -0.516 | 0.697 | -0.741 | 0.459 | 0.597 | (0.15, 2.34) | 0.561 | 1 | 0.454 | No |

**Supplementary Table S5. Interaction between rs2394445 and clinical characteristics**

TC: total cholesterol; TG: total triglyceride; HDL-C: high-density lipoprotein cholesterol;

LDL-C: low-density lipoprotein cholesterol;

BMI: body mass index; WHtR: waist-to-height ratio.
